# Biallelic, Selectable, Knock-in Targeting of CCR5 *via* CRISPR-Cas9 Mediated Homology Directed Repair Inhibits HIV-1 Replication

**DOI:** 10.3389/fimmu.2022.821190

**Published:** 2022-03-21

**Authors:** Stefan H. Scheller, Yasmine Rashad, Fayez M. Saleh, Kurtis A. Willingham, Antonia Reilich, Dong Lin, Reza Izadpanah, Eckhard U. Alt, Stephen E. Braun

**Affiliations:** ^1^Applied Stem Cell Laboratory, Medicine/Heart and Vascular Institute, Tulane University Health Sciences Center, New Orleans, LA, United States; ^2^Department of Cardiology and Angiology, Faculty of Medicine, Otto-von-Guericke-University Magdeburg, Magdeburg, Germany; ^3^Division of Immunology, Tulane National Primate Research Center, Tulane University School of Medicine, Covington, LA, United States; ^4^Department of Medical Microbiology, Faculty of Medicine, University of Tabuk, Tabuk, Saudi Arabia; ^5^Department of Surgery, Tulane University Health Science Center, New Orleans, LA, United States; ^6^Isar Klinikum Munich, Munich, Germany; ^7^Department of Pharmacology, Tulane University Health Science Center, New Orleans, LA, United States

**Keywords:** CCR5, co-receptor of human immunodeficiency virus type 1 (HIV-1),adipose-derived stem cells (ASCs), CRISPR-Cas9, biallelic mutations, homology directed repair (HDR)

## Abstract

Transplanting HIV-1 positive patients with hematopoietic stem cells homozygous for a 32 bp deletion in the chemokine receptor type 5 (CCR5) gene resulted in a loss of detectable HIV-1, suggesting genetically disrupting CCR5 is a promising approach for HIV-1 cure. Targeting the CCR5-locus with CRISPR-Cas9 was shown to decrease the amount of CCR5 expression and HIV-1 susceptibility *in vitro* as well as *in vivo*. Still, only the individuals homozygous for the CCR5-Δ32 frameshift mutation confer complete resistance to HIV-1 infection. In this study we introduce a mechanism to target CCR5 and efficiently select for cells with biallelic frameshift insertion, using CRISPR-Cas9 mediated homology directed repair (HDR). We hypothesized that cells harboring two different selectable markers (double positive), each in one allele of the CCR5 locus, would carry a frameshift mutation in both alleles, lack CCR5 expression and resist HIV-1 infection. Inducing double-stranded breaks (DSB) *via* CRISPR-Cas9 leads to HDR and integration of a donor plasmid. Double-positive cells were selected *via* fluorescence-activated cell sorting (FACS), and CCR5 was analyzed genetically, phenotypically, and functionally. Targeted and selected populations showed a very high frequency of mutations and a drastic reduction in CCR5 surface expression. Most importantly, double-positive cells displayed potent inhibition to HIV-1 infection. Taken together, we show that targeting cells *via* CRISPR-Cas9 mediated HDR enables efficient selection of mutant cells that are deficient for CCR5 and highly resistant to HIV-1 infection.

## Introduction

Since the discovery of the human immunodeficiency virus (HIV) in 1983, the introduction of antiretroviral drug therapy (ART) has turned classically acute HIV infection into a chronic condition. Although ART effectively inhibits HIV replication and disease progression, it does not eliminate the virus (1). Consequently, viral load rebounds when ART is removed and lifelong therapy is required to control viral reactivation and replication (2). Hence, research continues to find a cure for HIV (3, 4).

One potential target is CCR5, a major co-receptor utilized by HIV-1 for cellular entry (5, 6). High levels of CCR5 expression are found in CD4^+^ T cells and specific myeloid cell types, which become depleted during HIV-1 infection. A small population of individuals are resistant to HIV-1 infection and were found to be homozygous for a naturally occurring 32 bp deletion (CCR5 Δ32) mutation that inhibits CCR5 surface expression and confers resistance to infection by HIV-1 (7–9). So far, population studies were not able to identify deleterious effects of CCR5 Δ32, even in the case of homozygosity, indicating genetic disruption of CCR5 is not associated with major health risks (10). However, subtle changes like increased susceptibility to certain flaviviruses have been reported (11, 12). Based on this natural resistance, cancer patients with an active HIV-1 infection received allogeneic hematopoietic stem cell transplantation (HSCT ) from donors homozygous for CCR5 Δ32 (13). In two cases, individuals have been reported with a functional cure from HIV-1 infection (14, 15). Thus, CCR5 Δ32 has been identified as a promising target for curing HIV-1 (16–18). As not all patients have suitable donors, additional approaches are necessary to create a widespread applicable cure for HIV-1 (4). Several approaches for creating a CCR5 deficiency by disrupting its genomic locus have been undertaken, some of them even tested in clinical trials (17–21).

In these successful cases, cord-blood derived hematopoietic stem cells were used as the regenerative cell population (22–24). Besides HSC, other types of human stem cells have been characterized for their hematopoietic potential, such as induced pluripotent stem cells (iPSC), embryonic stem cells (ESC ) and mesenchymal stem cells (MSC ) (25–30). Adipose tissue derived stem cells (ASCs) are MSC resident within the heterogeneous group of cells within the stromal vascular fraction (SVF), which are more specifically labeled as vascular-associated, pluripotent stem (vaPS) cells (31). They have been shown to be differentiable into cells of all three germ layers including hematopoietic lineage and infectable by HIV-1 *in vitro*, which makes them a potential regenerative source for the blood cell pool depleted during HIV-1 infection (26, 29). In support of this hypothesis transplantation of MSCs isolated from mouse adipose tissue has been shown to efficiently rescue lethally irradiated mice from death as well as resulting in reconstitution of the major hematopoietic lineage (32). In addition, intravenous MSC transfusions in HIV-1 infected nonimmune responder (NIR) individuals showed a significant increase in their naive and central memory CD4^+^ T-cell counts, but it is unclear if the transplanted MSCs themselves filled up the T-cell compartment (33, 34). MSCs are attracted to latent HIV-1-infected cells and enable virus reactivation from the latent reservoir, so this cell type may play a role in HIV-1 infection beyond the ability to act as progeny for depleted cell pools (35). Because of its abundant availability within the easily accessible white fatty tissue, ASCs have become an attractive source of regenerative cells (31, 36, 37). After isolation from the patient‘s own adipose tissue, ASCs are suitable for immediate transplantation or expansion, genetic modification, and autologous transplantation. Thus, employing autologous ASCs may bypass bone marrow stromal/stem cells (BMSC) and HSC associated obstacles like complex isolation processes and unsatisfactory cell yields (38, 39). Additionally, autologous transplantation avoids the need for HLA matching and health risks associated with allogeneic stem cell transplantation (40).

Besides many others, CRISPR- Cas9 is a promising gene editing tool enabling the induction of precise changes in the human genome by creating double stranded breaks (DSB) (41–44). The resulting mutations are mediated by two major DNA repair mechanisms: non homologous end joining (NHEJ) and homology directed repair (HDR). While NHEJ mostly creates insertions or deletions (InDels) of smaller size, HDR fixes DSB *via* recombination of homologous sequences. This allows for the integration of foreign sequences into the targeted locus when located within the homology domain (HD) (45–47). Successful gene editing of CCR5 using CRISPR-Cas9 has been reported in a broad variety of studies (23, 27, 48–52). Yet, genomic changes induced by the CRISPR system, especially HDR, exhibit limitations in efficiency and creating predictable genotype outcomes has remained challenging (53–55). Because CCR5 heterozygosity is associated with postponed progression to AIDS in infected patients, only the individuals homozygous for the CCR5-Δ32 frameshift mutation, which lack all CCR5 expression, confer complete resistance to HIV-1 infection (7). Therefore, the efficiency of CRISPR mutations is important for a curative therapy. For inhibition of viral replication in an individual’s body, mathematical modeling estimates the fraction of susceptive cells needed to be made refractory to infection lies above 75 - 87.5% (56–58). Consequently, gene edited stem cells used for transplantation should have the highest possible or ideally complete mutational status. By this means it could be possible to provide the patient with a sufficient pool of resistant cells to regenerate the blood system under the selective pressure of HIV-1 infection (59, 60).

In this study, we test an approach for targeting the CCR5 gene and selecting biallelic frameshift mutated cells to create populations consisting of completely CCR5 deficient cells. We hypothesized that integrating two different fluorescent markers using CRISPR-Cas9 mediated HDR induces large frameshift mutations, which subsequently would result in a definite disruption of the CCR5 gene . Cells that constitutively express both fluorescent markers (double positive) could thus be recognized with a bi allelic frameshift mutation and selected rapidly and in large quantities using FACS. We disrupted the CCR5 gene in four different cell types, including human ASCs, and showed loss of CCR5 expression and inhibition of HIV-1 replication.

## Materials and Methods

### Cas9 and gRNA Targeting Plasmids

The pX330-U6-Chimeric_BB-CBh-hSpCas9 expressing a humanized S. pyogenes Cas9 (hSpCas9) from Dr. Feng Zhang (Plasmid #42230, Addgene, Watertown, MA) served as a scaffold. The guide RNAs (gRNAs) were synthesized as single-stranded synthetic oligonucleotides (IDT, Coralville, IA) and the complementary oligonucleotides were annealed to generate double-stranded DNA fragments with 5’ ACCG and 5’ AAAC overhangs (61). To generate the Cas9-gRNA expressing plasmids (**Figure S1A**), the gRNA linker was ligated into the Cas9 plasmids after BbSI digestion (New England Biolabs (NEB), Ipswich, MA, Cat. # R3539) using T4 DNA Ligase (NEB, Cat. # M0202). Two plasmids, pDONOR-tagBFP-PSM-EGFP (Addgene #100603) and pDONOR-tagBFP-PSM-dTOMATO (Addgene #100604), kindly provided by Jens Schwamborn, served as a template for the donor plasmids. To generate the homology domains (HD), we used a two-step PCR approach to insert the gRNA target sequence at the extremities of the HD into the donor plasmids. Cloning of the final donor plasmids (pDs) (**Figure S1B**) was carried out using Gibson Assembly Cloning Kit (NEB, Cat. # E5510S) in a modified fashion of the protocol published by Jarazo et al. (62). All plasmids were screened for correct formation *via* sanger sequencing.

### Cell Culture and Differentiation Assays

TZM-bl cell line (Cat. #ARP5011) and human T-cell Lymphoma Jurkat (E6-1) cell line (Cat. #ARP-177) were obtained through the NIH HIV Reagent Program. TZM-bl and HEK-293FT (Invitrogen, Carlsbad, CA, Cat. #R70007) cell lines and were cultured in low glucose DMEM medium (Invitrogen, Cat. #11885084) supplemented with 10% fetal bovine serum (FBS) (Invitrogen, Cat. #26140079) and 1% Penicillin/Streptomycin (Invitrogen, Cat. #15140163) at 37°C and 5% CO_2_, while Jurkat cells were cultured in RPMI-1640 (Invitrogen, Cat. #61870036) plus 10% FBS and 1% Penicillin/Streptomycin.

ASCs were isolated from fresh human lipoaspirate samples collected from healthy individuals during surgical procedures. The collection of all human tissue samples was done with the patient’s consent in an anonymized fashion and approved by the Institutional Review Board (IRB) of Tulane University, School of Medicine, New Orleans, Louisiana (IRB protocol #168758). Isolation was performed employing a Transpose^®^ RT Tissue Processing Unit (InGeneron, Houston, TX) according to the manufacturer’s instructions (36). These cells, designated as a passage 0 (P_0_), were then plated at a density of max. 5000/cm^2^ in alpha-MEM (Invitrogen, Cat. #12571063), supplemented with 20% FBS in standard cell culture conditions. For the following experiments, only low passage cells (P_1-3_) were used. To test isolated ASCs for multilineage differentiation potential adipogenic and osteogenic differentiation was performed as previously described (39, 63). For chondrogenic differentiation, cells were plated as a micromass culture using StemPro™ Chondrogenesis Differentiation Kit (Invitrogen, Cat. #A1007101) (**Figure S2**).

### Transfection

Since 293FT and TZM-bl are considered easily transfectable cell lines, lipofection was the transfection method of choice. Lipofectamine 2000 (Invitrogen Cat. # 11668500) was used according to the manufacturer’s instructions. Jurkat T-cells and especially primary ASCs are considered difficult to transfect. Due to the need of transfecting four different, largely sized (8.4kb/11.7kb) vector plasmids, the Neon Electroporation System (Invitrogen Cat. # MPK5000) was found to be the most promising approach. The system was used according to the manufacturer’s instructions. Briefly, 1 µg of each plasmid (4 µg total) and 5 x 10^5^ cells were brought into suspension in a 10 µl neon tip. After optimization, ASCs were transfected with 1 pulse at 1500V for 20 ms or 3 pulses at 1400V for 10 ms; while Jurkat cells were transfected with 1 pulse at 1200 to 1300V for 30 ms. The cells were immediately transferred to prewarmed antibiotic-free media. The viability and transfection efficiency was estimated by trypan blue staining and fluorescence microscopy or flow cytometry.

### Fluorescent Activated Cell Sorting

In cells with low transfection efficiency, cells underwent sorting for positive transfection 48 hrs post transfection (p.T.). To sort cells for constitutive expression of both fluorescent positive selection marker (PSM), and lacking the negative selection module (NSM) BFP expression (EGFP**^+^**,dTomato**^+^**,BFP**^-^**) 14 days post transfection, cells were suspended in PBS with 2% fetal bovine serum, 1% Penicillin/streptomycin. Sorting in all cell types was conducted with the same defined gating hierarchy: First FSC-A/SSC-A was used to identify the isoform cell population of interest and exclude debris. Secondly, FSC-W/FSC-H and SSC-W/SSC-H gating helped exclude doublets. Cells were then gated for BFP negativity. In the last gating step, we identified the EGFP and dTomato positive cells. For compensation reasons and identifying positive populations, negative and singly positive control groups were applied. All cell analysis and sorting steps were performed with a BD FACSAria III at the Cell Analysis & Immunology Core Facility at Louisiana Cancer Research Center. EGFP, dTomato and BFP were assessed by using FITC, PE and V450 filter sets respectively. Data was analyzed using FlowJo Software v_10.6.1 (FlowJo LLC, Ashland, Oregon).

### Immunophenotyping

ASCs were characterized by immunostaining with differently fluorescent labeled antibodies for mesenchymal and hematopoietic stem cell markers: CD90-APC, CD49b-APC, CD44-FITC, CD105-PE/APC, CCR5-APC, CD4-eFluor, CD34-PE, CD14-PE-Cy5, CD45-PE and CD68-PE (BD Biosciences, Franklin Lakes, NJ) (26). Analogously TZM-bl and Jurkat-T-cells were stained for CCR5 (CD195, BD Bioscience, Cat. #556903) surface expression using standard staining methods. If positive and negative cells were not distinguishable as two separate populations, Overton histogram subtraction technique was utilized for determining the fraction of positive cells (64).

### T7EI- Assay

To assess the cleavage efficiency of CRISPR-Cas9 targeted cells, T7 endonuclease I (T7EI) mismatch cleavage assay (IDT, Coralville, IA) was employed. Genomic DNA was isolated using the Mammalian Genomic DNA Miniprep Kit (Sigma-Aldrich). A PCR was performed, spanning a segment of 590 bp surrounding the targeted region using T7EI primer pair (**Table S1**). T7EI- Assay was carried out according to manufacturer’s instructions and the product was visualized *via* TBE Gel electrophoresis. Cleavage efficiency [Fcut = (b + c)/(a + b + c)] was calculated by measuring the band intensity of the undigested PCR product (a) and each cleavage product (b and c) with ImageJ_1.52a software (Rasband, W.S., ImageJ, U. S. National Institutes of Health, Bethesda, Maryland, USA). It should be noted, however, that the mutation rates determined by this strategy underestimate actual mutation frequency since small insertions or deletions (InDels) are not detected.

### qPCR for Assessment of Integration and Quantification of the Frequency of Mutation

**Figure 1:**
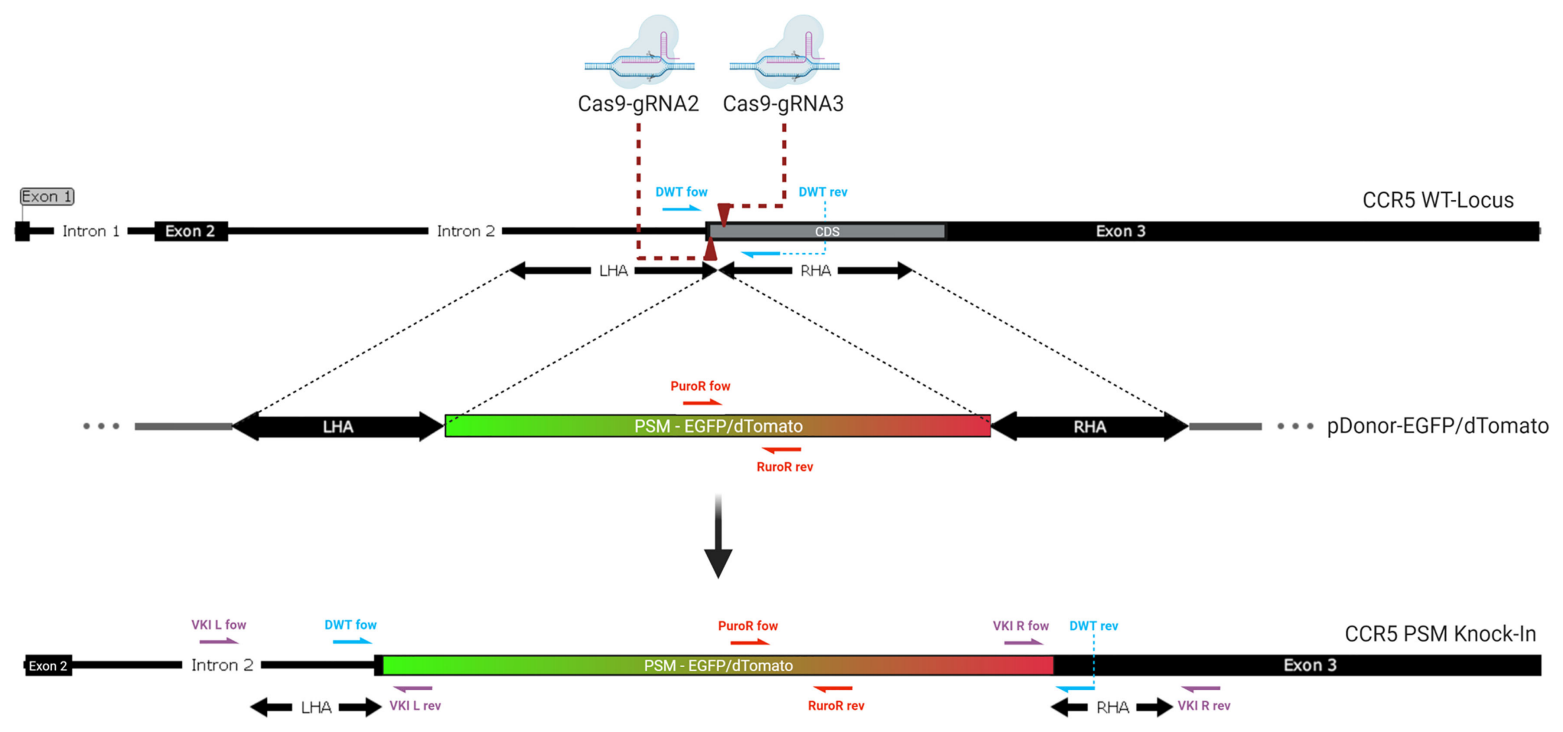
Genomic integration of the donor. Two Cas9-gRNA ribonucleoproteins are directed against closely adjacent sites on the coding sequence (CDS) at the beginning of Exon 3 of the CCR5 gene (dark red arrows). Double stranded breaks lead to integration of a donor template with homologous sequences matching the region surrounding the DSBs. The positive selection module (PSM), a non-homologous graft sequence, which lies between the left (LHA) and right homology arm (RHA) is being integrated into the CCR5 gene as homologous recombination occurs. It contains an expression cassette for either EGFP or dTomato. The knock-in of this long functional sequence creates a massive frameshift mutation, disrupting the CCR5 gene. Half arrows display the location of the primer pairs used for genomic qPCR. (violett: VKI; red: PuroR; blue: DWT).

Targeted and sorted populations were screened for integration of the PSM and disruption of the WT locus by qPCR using four primer pairs (**Figure 1** and **Table S1**). The vector knock-in (VKI) left and right primers span the left and right junction from the PSM across the Homology Arms to Intron 2 or Exon 3 respectively and positive amplification validates VKI of the PSM. The PuroR primer pair span the puromycin resistance gene and detect the either episomal or integrated PSM plasmid present in the population. The DWT (detecting the wildtype) primer pair spans the target site. Integration of the ~4200bp PSM inhibits amplification of the DWT by increasing the distance between the primer pair and therefore indicates integration in the CCR5 locus.

Since this study aims to inhibit the expression of CCR5 by disrupting its genomic locus, the amount of remaining and potentially functional WT Locus is of significant interest. Real-time quantitative PCR is considered a semiquantitative method of measuring a nucleic load, only allowing comparisons within signals detected with the same primer pairs on different genomic samples.

To estimate the percentage of undisrupted CCR5 loci in a targeted population, a standard curve was created from wild type genomic DNA (gDNA) mixed with gDNA derived from a single ASC clone carrying a complete biallelic knock-in of the PSM at different ratios (**Figure S4A**). It was thus possible to infer the percentage of DWT from the ΔCt *via* a linear regression by using the slope of the standard curve. Due to the logarithmic nature of qPCR, a correlation between the Ct value and the amount of wild-type locus exists only in the lower percentage ranges (**Figure S4B**). In a population with low levels of integration, minor ΔCt alterations would reflect increased levels of calculated alleles not carrying a knock-in. Consequently, the percentage of CCR5 alleles not carrying a knock-in (DWT) is only valid for populations in which ΔCt lies within a certain range (<20%), valid for reflecting mutational status. Thus this qPCR approach is rather an approximation of disruption by integration than an exact determination. Since CRISPR-Cas9 induced DSB themselves (without the occurrence of HDR) may lead to genetic disruption by InDels, a T7EI assay has been performed on the DWT amplicon. Therefore the mutational frequency is calculated as:


[% Mutant Alleles=% HDR+(% DWT × % InDel)]

with [% HDR = 1 – % DWT]. For all populations not targeted with HDR and with DWT ΔC_t_ signals above ranges considered valid, only the InDel frequency was taken into account for calculating the total remaining WT Alleles.

### HIV-1 Infection and Luciferase Reporter Gene Assay

TZM-bl cells express a firefly luciferase (Luc) reporter gene based on HIV-1 infection and HIV-1-Tat expression (50). Viral inhibition assay was performed by infecting 30,000 TZM-bl cells with R5-Tropic HIV-1_BaL_ virus (NIH HIV Reagent Program, Division of AIDS, NIAID, NIH, Manassas, VA Cat. # ARP-510) (titrated to induce >100,000 RLU Luciferase activity) for 3 hours (65). Cells were washed and cultured for 48 hours before lysis with 1x Reporter Lysis Buffer (Promega, Madison, Wisconsin, Cat. # E4030). The cell lysate was centrifuged at 20000 × g for 10 min and 20 μl of the supernatant were mixed with 100 μl of Luciferase Assay Reagent (Promega, Madison, Wisconsin Cat. # E4030) immediately before measuring luminescence with a Lumat LB 9507 (Berthold Technologies GmbH & Co. KG, Bad Wildbad, Germany). The protein concentration, as measured with NanoDrop™ 2000 spectrophotometer (ThermoFisher Cat. # ND-2000), was used to normalize the RLU/ug for each population in quadruplicates.

### Statistical Analysis

Results were presented graphically using GraphpadPrism8.1 (GraphPad Software Inc., San Diego, CA) or Excel 14.0.7265.5000 (Microsoft, Redmond, WA). Where meaningful, data is summarized using descriptive statistics such as mean, and standard deviation. Two-tailed student’s t-test and Wilcoxon matched-pairs signed-rank test were used as statistical methods and are referred to in combination with the presentation of the data . The study hypotheses were tested at a 5% level of significance throughout the analysis.

## Results

### Identification of the Most Efficient gRNAs and Their Combinations

Targeting CCR5 *via* CRISPR-Cas9 induced HDR requires three components: the Cas9 nuclease or its encoding sequence, a single guide RNA (gRNA) and a donor which will be integrated into the targeted locus. We hypothesized if cleavage occurs slightly downstream of the beginning of the coding sequence (CDS), mutations may be more likely to inhibit the expression of any functional CCR5 product (**Figure 1**). We used CRISPOR Version 4.99 (http://crispor.tefor.net/) to identify potential gRNAs and CasOFFfinder 2.4 (http://www.rgenome.net/cas-offinder/) to identify potential off-target sites (**Table S2**). gRNAs were excluded if they only displayed two or less mismatches with an off-target site, while three or more mismatches were considered acceptable. The four gRNAs with the highest predicted efficiency and least probability for off target effects were selected for screening (**Table S1**). The aim of this study is to create a selectable, biallelic, frameshift mutation, *via* the integration of two different fluorescent selectable markers, one in each allele. Arias-Fuenzalida et al. published a mechanism for fluorescence guided biallelic HDR targeting selection, using CRISPR-Cas9 and two donor plasmids to induce a single nucleotide change exclusively on one allele, linked to early-onset Parkinson’s disease (54). We assumed the integration of fluorescent markers would also act as a large frameshift mutation and therefore efficiently disrupt the CCR5 gene. In our previous study, we showed increased efficiency for biallelic mutations in the CCR5 locus using multiple guide RNAs in ASCs (55). This approach has previously been tested for ZFNs and TALENs and achieved predictable deletions (49). Hence two pCas9-gRNAs were selected and implemented in this study.

To determine whether dual or single targeting is more efficient in this target site and which combination of gRNAs displays the highest cleavage efficiency, four gRNAs (gRNA 1, 2, 3 and 4) as well as their combinations were screened. Transfections and T7EI Assays were performed in triplicates in 293FT Cells (**Figure S3**). gRNA3 showed to induce the highest amount of genomic alterations (32.3 ± 1.7% SD; n=3), followed by gRNA2. gRNA1 displayed only very weak activity and gRNA4 no activity at all. With (44.8 ± 18.0% SD; n=3), the combination of 2 + 3 showed the overall highest cleavage efficiency. Still, in this specific setup, the difference in mutational events by targeting with two gRNAs (2 + 3) rather than one (3) was not found to be statistically significant (p=0.15) as determined by using a two-tailed student’s t-test.

### Transfection of pDonor-EGFP/dTomato and pCas9-gRNA Together Leads to Constitutive Expression of Both Fluorescent Selectable Markers

Either an EGFP or dTomato encoding sequence functioned as a positive selection marker (PSM) selectable by flow cytometry for a successful knock-in. DSB induced HDR is often found to be a very inefficient process. Multiple strategies to increase the integration rate have been incorporated into the donor design. First, the length of the homology arms has been shown to have a substantial impact on the integration efficiency (66). Second, linear donors were found to display higher integration rates than circular plasmids. However linear DNA is subject to relatively fast degradation which might limit the amount of donor available for integration. Therefore, gRNAs were integrated into the donor plasmids for linearization in presence of Cas9 activity (66). Third, minimizing the replaced sequence surrounding the DSB (66). Thus, the interior ends of the homology arms include the primary cutting sides of the Cas9. To prevent cleavage activity within the Homology Domain, the protospacer adjacent motif (PAM), the 5’.NGG.3’ sequence of gRNA 2 and 3, was changed to 5’.NCC.3’ so it will not be recognized by the Cas9-gRNA complex. All modifications listed above were integrated into the homology arms by designing specific primers (**Table S1**).

**Figure 2:**
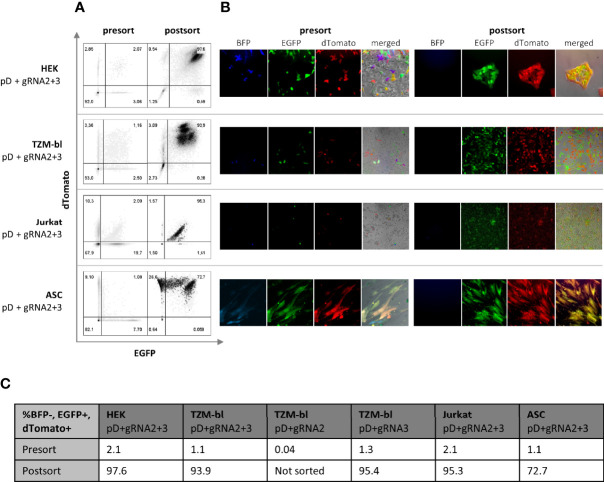
Selection of double positive (BFP-, EGFP+, dTomato+) cells *via* FACS. Four different cell types (HEK, TZMbl, Jurkat and ASC) have been transfected with both pDonor and both pgRNA-Cas9 14 days prior to sorting. To compare dual to single targeting, TZMbl have been transfected with both pDonors and single pgRNA-Cas9. **(A)** Cells were sorted for BFP negativity (not shown) and dual positivity for EGFP and dTomato (presort). The sorted population was reanalyzed immediately and 7 days later, prior to conducting the experiments (postsort) **(B)** Flourescent microscopy of cells 14 day post transfection (presort) and 7 days after sorting for double positivity (postsort) **(C)** Quantification of BFP-, EGFP+ and dTomanto+ cells before and 7 days after sorting. To compare dual to single targeting, TZMbl have additionally been transfected with both pDonors and single pCas9-gRNA (pD+gRNA3/pD+gRNA2).

In total, four different cell types (293FT, TZM-bl, ASCs and Jurkat T-cells) have been employed for testing the constructs. pDonor and pCas9-gRNA were delivered either by using Lipofectamine 2000 (293FT and TZM-bl) or the Neon Electroporation System (Jurkat T-cells and ASCs). HEK 293FT c ells showed high transfectability (>90% estimated by microscopy, data not shown); however, TZM-bl, Jurkat, and ASCs did not show as high transfection efficiencies (25.0-32.9% TZM-bl; 23.6% Jurkat; 37.9% ASC; assessed by flow cytometry, data not shown). Hence, TZM-bl, Jurkat and ASCs were sorted for positive transfection 48 hr post transfection (p.T.). Additionally, every transfection included the pCas9-gRNA only transfected comparison group, a negative, as well as a pDonor only control. Transfecting the pDonor alone allowed us to estimate the time until the transient expression of fluorescent marker subsides due to plasmid degradation as observed by fluorescent microscopy. After 14 days, control groups in all cell types lost their fluorescent signal (data not shown). Fluorescence displayed beyond this point in time was expected to be subject to constitutive expression due to the integration of the selectable marker into the genome. Consequently, sorting for constitutive expression of both PSMs (dTomato and EGFP) and absence of the NSM (BFP) was carried out on day 14 p.T. (**Figures 2A, B**). Using two sgRNAs, the frequency of double-positive cells was found to be between 1-2% across all cell types. Testing single targeting in TZM-bls induced comparable frequencies (1.3%) with gRNA3, while gRNA2 induced almost no constitutive expression (0.04%) (**Figures 2A, C**). To ensure a correct and stable expression pattern, the sorted population was reanalyzed directly and prior to conducting downstream experiments.

### Targeting-Selecting Enables the Selection of Genetically Disrupted Cells

**Figure 3:**
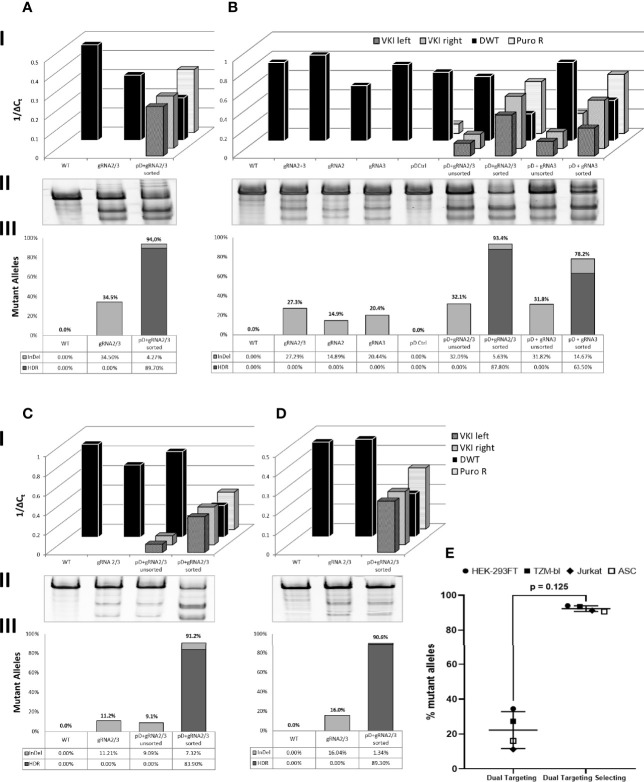
Quantitative analysis of genomic changes in target site. Dually targeted and selected cells (pD + gRNA2/3 sorted) were compared to WT and cells dually targeted with gRNA2/3. Additionally the genomic profile of targeted populations before sorting for dual positivity (pD+gRNA2/3 unsorted) was analyzed in **(B, C)** B also includes comparison groups targeted and target-selected with single gRNAs. I: Real Time quantitative PCR of total PSM (PuroR), the integrated PSM (VKI left/right) and the CCR5 Locus not carrying a knock-in (DWT). qPCR r esults are presented inversely (1/ΔC_t_), so a high and a low genomic load are represented by a tall and a low bar respectively. II: T7EI-Assay of the DWT Amplicon. III: Calculation of the Fraction of Mutant Alleles, carrying a knock-in (HDR) or an InDel (InDel): [%Mutant Alleles = HDR + ((1-HDR)*InDel)]. [HDR = 1 - DWT]. DWT, the fraction of alleles not carrying a knock-in, is calculated through linear regression of the ΔCt (DWT) on the standard curve as described above. InDel frequency was calculated as previously mentioned. **(A)** HEK 293FT. **(B)** TZM-bl. **(C)** Jurkat. **(D)** ASC. **(E)** Mean fraction of mutant alleles across all cell types in double positive, dual targeting selecting group (pDonor-EGFP/dTomato + pCas9-gRNA2/3) compared to dual targeted control group (pCas9-gRNA2/3). Error bars show ± SD. Statistical analysis was performed using Wilcoxon matched-pairs signed rank test.

To quantify integration and non-integration of the PSM, real time quantitative PCR was employed (**Figure 3**, row I). In all four cell types, dually targeted and selected cells were compared to WT cells and a dually CRISPR-Cas9 targeted control group (**Figure 3A–D**). WT cells and pCas9-gRNA only transfected control groups did not show any PuroR or VKI signal. PuroR was detectable in all groups transfected with pDonor, but the transfection of the donor alone (pD Ctrl) did not lead to any integration (VKI). VKI was only detectable in groups generating the DSB (transfected with pDonor and pCas9-gRNA), indicating only simultaneous transfection of all plasmids leads to integration of the Donor. To ensure that both CCR5 alleles are targeted, we included two pDonor plasmids with either EGFP or dTomato fluorescent markers. After selecting the pDonor + pCas9-gRNA transfected groups for double positive cells, the frequency of PuroR and both VKI increased compared to the corresponding unsorted group in TZM-bls and Jurkats (**Figures 3B, C**). More importantly, sorting for double positivity leads to a definite reduction of the CCR5 wild-type allele (DWT). All other control groups (WT, pCas9-gRNA alone, pD Ctrl, unsorted) display almost equal DWT signals, differing by less than one ΔCt within each cell type. Only the targeted and sorted populations had DWT in a range low enough to infer the fraction of alleles not carrying a knock-in (**Figure 3**, row III). T7EI Assay was performed on the DWT amplicon to quantify the InDel frequency within the alleles not carrying a knock-in (**Figure 3**, row II). WT and pD control do not display cleaved fragments. All groups transfected with pCas9-gRNA show cleavage activity with band sizes to the corresponding gRNAs transfected. Targeting with two gRNAs showed a mean mutational activity of 22.3% (± 10.6%; n=4) (**Figure 3E**), which is found to differ between the different cell types (**Figures 3A-D**). In TZM-bls (**Figure 3B**), two gRNAs (27.3%) show slightly higher cleavage efficiency compared to single gRNAs (14.9 – 20.4%) coinciding with the findings in HEK293FT cells (**Figure S3**). Additionally in TZM-bls and Jurkats (**Figures 3B, C**) sorted groups show higher InDel frequencies within the fraction of alleles without recombination (DWT) than their unsorted counterparts as detected by the surveyor Assay (**Figure 3**, row II).

To calculate the total fraction of mutant alleles for the sorted populations, disruption by HDR and InDel frequency were added (**Figure 3**, row III). Assuming all WT, comparison and unsorted groups do not carry any (detectable) KI, indicated by their DWT signal, only the InDel frequency was taken into account for calculating the mutational frequency. Across all cell types (**Figures 3A–D**), dually targeted and selected (double positive) populations showed consistently high mutation rates of averaging 92.4% (± 1.6%; n=4) (**Figure 3E**), of which 87.7% (± 2.6%; n=4) were due to HDR. Double positive TZM-bls targeted with only gRNA3 and two donor-plasmids showed a mutational frequency of 78.2%.

**Figure 4:**
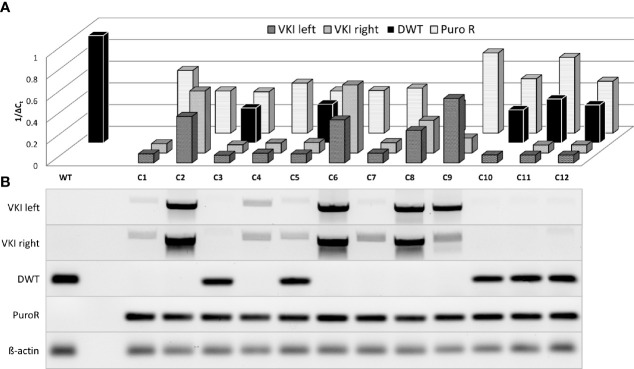
Semiquantitative analysis of genomic changes in dual target-selected, double positive ASC clones. ASCs were transfected with both pDonor and pgRNA-Cas9 (pD+gRNA2/3). 14 days p.T. twelve BFP-, EGFP+, dT+ clones were selected and separately cultured upon reaching enough cells for genomic analysis. **(A)** Real Time Quantitative PCR of sequences associated with HDR was carried out analogously to population analysis. **(B)** Agarose gel electrophoresis of PCR-Products.

To examine the genomic structure of an individual double-positive cell rather than a whole population, ASC clones were isolated and analyzed for homologous recombination, indicated by detectable VKI and PuroR signals (**Figure 4**). PuroR was positive in all clones. However, VKI signal intensity differed very widely between the different clones and even within one clone comparing VKI left and right. 7/12 clones display no DWT signal, suggesting integration inhibiting DWT amplification on both alleles. In 5/12 clones, DWT did amplify, but with a lower signal than the WT. Still, in these clones at least one allele can be expected to not carry a knock-in. The widely varying signal of the knock-in associated sequences, may be an indication for polyform integration mechanisms.

### CCR5 Surface Expression Is Abolished After Selection for Double Positive Cells

**Figure 5:**
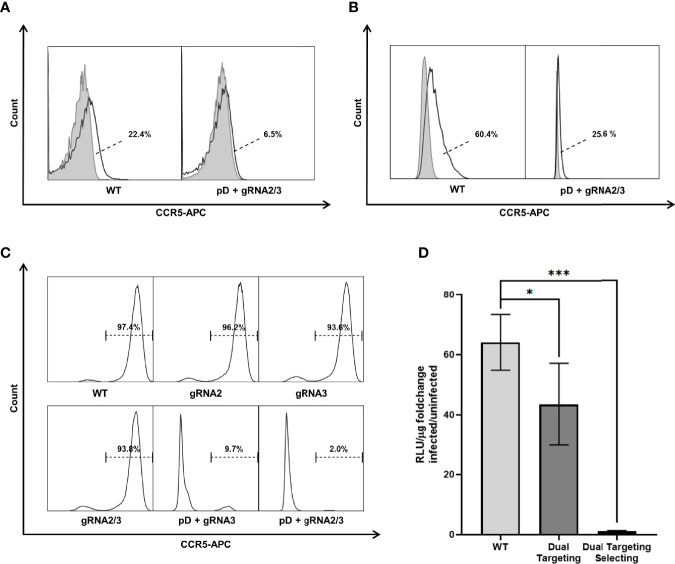
CCR5 surface expression was analyzed by flow cytometry in three different cell types. Untreated (WT) as well as dual targeted-selected (pD+gRNA2/3) populations were stained with an APC labeled Anti-CCR5 Antibody (black lined graph). Unstained negative samples (light gray filled graph) are shown to distinguish the CCR5 positive fraction. **(A)** Jurkat. **(B)** ASC. Fractions are presented next to the Graph. The dashed line points towards the CCR5 positive subset (white area) as calculated by overton subtraction technique. **(C)** TZM-bl includes additional differently targeted populations. Because of the easily distinguishable positive population, only the stained samples are shown. **(D)** HIV-1 infectivity of TZM-bl was measured by Luciferase Assay. Cell lysate was obtained 48h after infection with HIV-1_BaL_. Uninfected cells were used to measure the background signal. All experiments were carried out in quadruplicates. The results are presented as the fold change in luciferase activity by infection. Error bars show ± SD and significant changes are represented as p-values (*p < 0.05, ***p < 0.0005).

The genomic analysis of the targeted selected cells indicated substantial disruption of the CCR5 locus. The CCR5 receptor executes its function by being displayed on the host cell‘s surface. To investigate whether the genomic changes lead to a loss in CCR5 surface expression, TZM-bls, Jurkat T cells and ASCs were stained for CCR5 expression (**Figures 5A‐C**). Jurkat T cells and ASCs express low levels of CCR5 on their surface. Overton histogram subtraction technique was used to distinguish positive cells (64). However, dual targeting selection was able to reduce the fraction of CCR5 positive cells from 22.4% in WT Jurkat to 6.5% after targeting (**Figure 5A**). Similarly, a reduction of CCR5 positive cells from 60.4% to 25.6% was found in ASCs (**Figure 5B** ). In TZM-bl cells, CCR5 is expressed in 97.4% of WT cells (**Figure 5C**). Targeting with CRISPR-Cas9 alone using gRNA2, gRNA3 and dual targeting only lead to minor reduction in CCR5 expression. In contrast, targeting and selection of dual positive cells lead to a significant reduction of detectable surface CCR5 to 9.7% and 2.0% using one and two gRNAs respectively (**Figure 5C**).

### Double Positive TZM-bl Cells Show Low Infectability for HIV-1

For successful entry of the host cell, the HIV-1 particle binds first to CD4 and opens the CCR binding domain in the gp120 variable loop; then, binding to CCR5 forms an integration complex, which mediates fusion of the virion into the cell (67). To determine whether the mutations in CCR5 prevented infection, TZM-bls were exposed to HIV-1_BaL_ and infectability was assessed by Luciferase Assay (**Figure 5D**). WT TZM-bls show a significant increase in luciferase activity when challenged with HIV-1 (64.1 ± 8.0 fold increase; n=4) compared to uninfected control. The increase is reduced in cells dually targeted with CRISPR-Cas9 alone (43.5 ± 11.8 fold increase; n=4). Dually targeted and selected TZM-bls show almost no change in luciferase activity, when exposed to HIV-1 (1.2 ± 0.1 fold increase; n=4) compared to uninfected controls (**Figure 5D**). Consequently targeting with two gRNAs leads to a significant reduction in infectability (67.2 ± 17.6%; p=0.03) compared to WT cells but still leaves a high level of infection. In contrast, double positive TZM-bls show only a minor fraction of the infectability of WT Cells (1.9 ± 0.4%; p=0.00043) (**Figure 5D**).

### Targeting Selecting ASCs Leaves Regenerative Potential Unaltered

**Figure 6:**
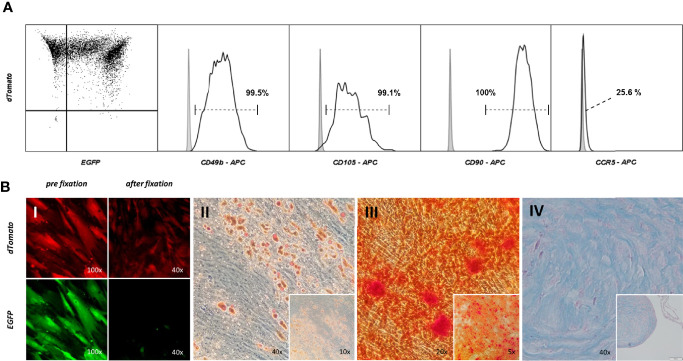
Targeting – selecting ASCs leaves regenerative potential unaltered. **(A)** Immunophenotypic analysis of cell surface profile of double positive ASCs. Cells were stained with fluorescent marker labeled antibodies for CD90, CD49b, CD105 and CCR5. The gray filled graph represents the signal of an unstained control, the black line the population stained with the corresponding antibody. **(B)** Multilineage differentiation Assay. Double positive ASCs were capable of adipo- (II), osteo- (III) and chondrogenic (IV) differentiation. Cells were checked for the correct fluorescent expression pattern. EGFP was washed out during formaldehyde fixation (I).

Analogue to testing ASCs prior to conducting experiments, it was investigated whether targeted-selected ASCs keep their multipotency characteristics. Roughly 70% of ASCs appeared to be double positive, leaving the remaining 30% only dTomato positive. Characteristic mesenchymal stem cell marker antigens were displayed on the cell’s surface (CD49b, CD105, CD90) (**Figure 6A**). The population was clearly negative for surface CCR5, unlike the WT ASCs which displayed a low but detectable signal when stained for CCR5. Targeted and s elected ASCs were capable of differentiating into adipogenic, osteogenic and chondrogenic lineages (**Figures 6B II-IV**) to the same extent as WT ASCs. Double positive cells consistently expressed EGFP and dTomato throughout differentiation but lost EGFP expression due to fixation and the staining process (**Figure 6B I**).

## Discussion

Among many other approaches to create a functional or sterilizing cure for HIV-1, the clinical success in the “Berlin Patient” has made genetically ablating CCR5 a focal point of research in this area (18, 19, 68, 69). New gene editing techniques, in particular CRISPR-Cas9, offer promising possibilities for targeted mutation of the CCR5 gene, but often the mutation rate falls short and not every mutation reliably leads to a functional disruption. Thus, after genetic modification, one obtains a diverse population of cells containing completely disrupted, partially disrupted, or undisrupted cells. Mathematical modeling estimates, for inhibition of viral replication in an individual, the fraction of cells (CD4^+^ T cells) refractory to infection needs to be above 75-87.5% (56–58). Another study suggests that only 10–20% CCR5 knock- out in CD34^+^ HSC would maintain CD4^+^ T cell counts >200 cells/μl after 10 years when the modified cells have a selective advantage (70). To achieve this proportion of modified cells in patients, the generation and transplantation of sufficient amounts of regenerative cells rendered resistant to HIV-1 infection is a prerequisite, especially when there is no previous cytoreduction by ablative chemotherapy. These cells would ideally be autologous, deficient of CCR5, and have complete regenerative potential. Many strategies have been investigated to increase knock- out efficiencies in CCR5 (23, 49, 50, 55, 71, 72). Besides increasing gene editing efficiency, one strategy is to select edited cells prior to transplantation (73). However, stem cell populations such as HSCs, iPSCs, and ASCs, which have been shown to differentiate into CCR5 expressing cells with hematopoietic characteristics, normally show only low to no expression of CCR5 (25–30). Thus, it is not possible to select CCR5 disruption in stem cells based on the absence of the receptor’s surface expression, but requires creating the selection based on another phenotype.

In this study we used CRISPR-Cas9 mediated homologous recombination to integrate two different fluorescent markers into the CCR5 gene, functioning as a large frameshift mutation and selectable marker. We hypothesized that this mechanism enables selection of bi allelic frameshift mutated cells based on the genotype which are deficient for CCR5. To completely eliminate CCR5 expression, as in individuals that are homozygous for Δ32, it is expected that both alleles in all cells would need to be edited (55). Instead of increasing the mutation efficiency or isolating single clones with the desired mutational status, we pursued a strategy enabling a bulk selection of cells with a genomic pattern likely to lead to complete disruption, an approach similar to introducing mono allelic single-nucleotide changes (54, 62). HDR, regardless of zygosity, is known to be a quite inefficient process (42, 53, 74). Therefore, we integrate a strategy of dual targeting to allow for selection of bi allelic mutational events (27, 49, 55). Not all biallelic mutations will be double positive (integration of both fluorescent markers, each on one allele), since 50% of biallelic recombination is expected to be with a single fluorescent marker (either EGFP or mCherry) in both alleles. Sorting for double positive cells ensure that both alleles have been targeted. In order to obtain a sufficient number of successfully edited cells, either a large population or expansion of the autologous regenerative cells would be necessary. Adipose tissue derived stem cells (ASCs) show ideal properties regarding isolation and expansion to support such strategies and represent a potential population for replenishing the immune cell compartment (26, 29, 32, 39). Consequently, we tested applicability in four different cell types, including CD4^+^ Jurkat T cells and ASCs.

Transfection of Cas9 and gRNA encoding vectors as well as donor plasmids with fluorescent selectable markers lead to integration of the donor and constitutive expression of double-positive fluorescent cells. Coinciding with previous findings (54, 62, 75), we were able to observe 1-2% double positive cells, depending on the cell type and gRNAs used. Performing one sorting step using FACS, we were able to create populations consisting of up to 97.6% constitutively double-positive cells in cell lines and 72.0% in ASCs. Genomic analysis revealed dually targeted and selected double-positive populations carry mutations in 92.3% (± 1.6%; n=4) of all alleles, of which the largest share are disrupted by HDR based on the standard curve. The residual WT CCR5 locus was also significantly reduced. This is a much higher mutational status than what we found or has been previously reported by knock-out studies using single or dual targeting with CRISPR-Cas9 without a selection system (23, 27, 48–51, 72, 76). Coinciding with our previous findings, targeting with two gRNAs displayed higher InDel frequencies than using a single gRNA. For example, single-targeted dual-positive TZM-bls showed a lower mutational frequency than dually targeted cells (78.2% vs. 93.4%). So dual targeting may lead to more thorough DSB formation and homologous recombination than using a single gRNA. Sorting for double-positive cells was shown to select cells the majority of which integrated the PSM (increased VKI) and thus had a disrupted CCR5 gene (decreased DWT). Even though sorting enabled selection of a population heavily disrupted in the CCR5 locus, it did not lead to complete elimination of cells with the wild-type allele. One explanation is the limitation in sorting a population with 100% double-positive cells; however, this cannot be completely responsible for the remaining alleles without integration. In the 12 double-positive clones, genomic DNA analysis shows a DWT signal in 5 of the 12 clones. Although DWT is clearly reduced in these clones compared to the wild type, it indicates at least one CCR5 allele remains unintegrated. Therefore, a certain fraction of double-positive cells do contain CCR5 without a knock-in, and thus the expression of one or both selectable markers does not originate from the CCR5 locus but has been integrated elsewhere. Consequently, double-positive populations cannot be considered as completely biallelic frameshift mutated. However, double positivity is a strong indicator for a high frequency of mutant alleles and disruption of the CCR5 gene. Still, to make precise statements about the extent and properties of bi allelic frameshift mutations in double positive cells, sequencing analysis in a large quantity of clones and/or determining the PSM integration site could be conducted.

More important than achieving complete mutational status was the question of whether targeting and selection is able to create populations deficient for a functional CCR5 receptor and therefore resistant to HIV-1 infection. Natural resistance is conferred by a 32 bp frameshift mutation in CCR5 leading to a premature stop codon after an additional 25 amino acids. Conventional targeting with CRISPR-Cas9 typically induces smaller size InDel mutations that result in minor insertions or deletions, missense, or nonsense mutation not severe enough to prevent expression of a functioning protein. In only a fraction of targeting events are frameshift mutations generated in both CCR5 alleles (55). Previous knock-out studies using conventional CRISPR-Cas9 targeting in TZM-bls were able to reduce the fraction of cells with CCR5 surface expression to as low as 49.2% (48, 50). Using a Lentiviral vector, a 41.2% or 33.3% reduction was possible (50, 72). Single and dual targeting in TZM-bls lead to InDel frequencies between 14.9 and 27.3% as assessed by surveyor assay (**Figure 3B**). However, the reduction in CCR5 surface expression was only as low as 1.2 - 3.8% (**Figure 5C**). This demonstrates that InDels alone do not create mutations severe enough to reliably inhibit CCR5 expression. In contrast, the integration of a large functional sequence including a promoter and terminator into the CCR5 coding sequence can act as a massive frameshift and inhibit proper transcription. We were able to detect a reduction of the measurable surface CCR5 from 97.4% in WT TZM-bl down to 2.0% in double-positive TZM-bls. Therefore in targeted and selected populations the drastic reduction in CCR5 expression correlates with the high frequency of mutation, predominantly caused by HDR. From this it can be concluded that large frameshifts induced by HDR lead to a functional disruption of CCR5 more reliably than it would be the case with InDel mutations alone. Although Jurkat and ASCs express CCR5 at low levels, a similarly significant reduction could be achieved by dual targeting selection in these cell types. Comparable knock-out studies were able to decrease infectability to roughly 40% when using conventional targeting and different transfection techniques in TZM-bls (48, 50). When challenged with HIV-1, double-positive TZM-bls infectability was inhibited 98.1% of WT TZM-bls level, compared to inhibition of 32.7% in the CRISPR-Cas9 dual targeting control. Additionally, the cultivation and modification of stem cells *in vitro*, even if performed carefully, involves a risk of loss in regenerative capacity. When characterized and tested for multilineage differentiation potential, double-positive ASCs showed the same properties as WT ASCs, suggesting this approach to be successfully applicable in stem cell based therapies. The extent of CCR5 surface expression on MSCs or ASCs in the literature is not clear (26, 29, 77–79), However, we were able to detect slight CCR5 expression on WT ASCs by immunophenotyping, which was eliminated in double-positive ASCs.

Often attempts to create populations with a high rate of a specific mutational pattern are bound to screening and expanding isolated clones. The main benefit associated with the presented strategy is the ability to select highly disrupted cells that are likely to be bi allelic frameshift mutations in a high throughput scale necessary for clinical applications. Although increased, we found the efficiency of HDR-based gene delivery and editing approaches to be a major limitation. We showed that a one-time selection of double-positive transfected cells *via* FACS enriched CCR5 HDR within the population of successfully transfected cells. Implementing alternative gene delivery methods and ways to increase integration could help yield larger quantities of double-positive cells prior to the sorting step. Fluorescent markers like EGFP and dTomato used in this study are beneficial for application in these preclinical proof-of-concept studies; however, they would be less useful in a clinical application creating the need for alternative selectable markers compatible for in-patients use (80). Previous studies have used puromycin selection to provide continuous selection pressure, eliminating cells which were not transfected or l ose the PSM due to plasmid degradation while subculturing (54). A two-drug selection mechanism would also select for bi allelic HDR; or alternatively, a single-drug mechanism would increase the number of edited cells, but not guarantee that both alleles have been targeted. Dual targeting and selection showed consistent outcomes across the tested cell types proving this concept to be reproducible in different scenarios. Our novel approach opens up new therapeutic options to cure patients from HIV-1 infection by using their own pool of regenerative cells. This would not only avoid the risks of lifelong antiretroviral therapy but also those associated with allogeneic transplantation strategies such as myeloablation and the obstacles of HLA matching.

## Conclusion

Taken together, this study provides proof-of-concept that selection for double-positive cells enriches for the integration of selectable markers into both CCR5 loci. It is thus possible to generate populations highly deficient for CCR5 and resistant to HIV-1 infection, representing an approach to bypass inefficiencies to reliably disrupt the CCR5 gene. The strategy doesn’t impair stem cell multilineage differentiation potential, opening up the possibility to be applied in stem cell based therapies. Combined with the application in adipose tissue derived stem cells, this is a novel strategy for the generation of sufficient amounts of HIV-1 resistant autologous regenerative cells. These could partly and repetitively reconstitute the immune system under the selective pressure of an HIV-1 infection and thus represent a possible approach for curing HIV-1.

## Data Availability Statement

The original contributions presented in the study are included in the article/**Supplementary Material**. Further inquiries can be directed to the corresponding authors.

## Ethics Statement

The collection of all human tissue samples was done with the patient’s consent in an anonymized fashion and approved by the Institutional Review Board (IRB) of Tulane University, School of Medicine, New Orleans, Louisiana (IRB protocol #168758).

## Author Contributions

Conceptualization: SHS, RI, EUA, SEB. Methodology: SHS, DL, RI, EUA, SEB. Investigation: SHS, YR, FMS, KAW, AR. Analysis: SHS, SEB. Writing—Original Draft: SHS, SEB. Writing—Review and Editing: SHS, YR, FMS, KAW, AR, RI, EUA, SEB. Visualization: SHS, SEB. Funding Acquisition: EUA, SEB. Supervision and Administration: SEB. All authors contributed to the article and approved the submitted version.

## Funding

These studies were funded by the Alliance for Cardiovascular Research, and supported by the National Center for Research Resources and the Office of Research Infrastructure Programs (ORIP) at the NIH through grant P51 OD011104 (TNPRC).

## Conflict of Interest

The authors declare that the research was conducted in the absence of any commercial or financial relationships that could be construed as a potential conflict of interest.

## Publisher’s Note

All claims expressed in this article are solely those of the authors and do not necessarily represent those of their affiliated organizations, or those of the publisher, the editors and the reviewers. Any product that may be evaluated in this article, or claim that may be made by its manufacturer, is not guaranteed or endorsed by the publisher.
