# Supplementary material for: Biallelic, Selectable, Knock-in Targeting of CCR5 via CRISPR-Cas9 Mediated Homology Directed Repair Inhibits HIV-1 Replication
Source: Front Immunol. 2022 Mar 21;13:821190. doi: 10.3389/fimmu.2022.821190 (PMC8978527; doi:10.3389/fimmu.2022.821190)
Supplement: Supplementary file 1 [file DataSheet_1.pdf]

Supplementary Material

Biallelic, Selectable, Knock-in Targeting of CCR5 via CRISPR-Cas9 Mediated Homology Directed Repair Inhibits HIV-1 Replication

Stefan H. Scheller, Yasmine Rashad, Fayez M. Saleh, Kurtis A. Willingham, Antonia Reilich, Dong Lin, Reza Izadpanah, Eckhard U. Alt, Stephen E. Braun

Table S1: Oligonucleotide sequences

Primer	Sequence (5'-3')	Function
**sgRNA1**	CTTTTTATTTATGCACAGGG	Single-guide cloning
**sgRNA2**	TAATAATTGATGTCATAGAT	Single guide cloning
**sgRNA3**	TGACATCAATTATTATACAT	Single guide cloning
**sgRNA4**	CTTCACATTGATTTTTTGGC	Single guide cloning
**LHA**	AAGCTTGGATCCCCTAGGTTTGACATCAATTATTATACATCGGTGAATG<br><br>TGACATCAATTATTATACATCGGTGAATGGGTATGATGCTTAGAACAG<br><br>CAGACTATCTTTCTAGGGTTTGATGTCATAGATTCCACTTGACAC	Cloning of the left homology arm for Gibson assembly, introduction of gRNA3 sequence and mutation of the NGG
**RHA**	ATGATTATCTTTCTAGGGTTATTATTATACATCCCAGCCCTGCC<br><br>CCGATGTATAATAATTGATGTCAAGAGCTACTGCAATTATTCAGGC<br><br>GCATACGCGTATACTAGGTTCCGATGTATAATAATTGATGTCAAGAGCT	Cloning of the right homology arm for Gibson assembly, introduction of gRNA3 sequence and mutation of the NGG

**hU6-F**	GAGGGCCTATTTCCCATGATT	Sequencing of the pCas9-gRNA
**Seq-RHA3`-junction (SeqM13)**	CAGGAAACAGCTATGAC	Sequencing of the Donor Plasmids
**Seq-LHA5`-junction**	CCTAGCAAACTGGGGCACAAGC	Sequencing of the Donor Plasmids
**T7EI**	GCTTCATTCACTCCATGGTGCTAT<br><br>CAAAGTCCCACTGGGCGG	T7EI Assay
**DWT**	GCATTCATGGAGGGCAACTAAA<br><br>GGCTGCGATTTGCTTCACATT	qPCR
**VKI left for**	AGGCTTCCCGCATTCAAAATC	qPCR
**VKI left rev Seq-LHA3`-junction**	CCTAAATGCACAGCGACGGA	qPCR/<br><br>Sequencing of the Donor Plasmids
**VKI right for Seq-RHA5`-junction**	ACCGATAAAACACATGCGTCA	qPCR/<br><br>Sequencing of the Donor Plasmids
**VKI right rev**	CAGTGCGTCATCCCAAGAGT	qPCR
**PuroR**	AGAGGAAGTCTTCTAACATGCGGT<br><br>AGAGTTCTTGCAGCTCGGTG	qPCR

Table S2. CRISPOR-Results

Sequence CTTTTTATTTATGCACAGGGTGGAACAAGATGGATTATCAAGTGTCAAGTCCAATCTATGACATCAATTATTATACATCGGAGCCCTGCC
AAAAAATCAATGTGAAGCAAATCGCAGCCCGCCTCCTGCCTCCGCTCTACTCACTGGTGTTCATCTTTGGTTTTGTGGGCAACATGCTG
GTCATCCTCATCCTGATAAACTGCAAAAGGCTGAAGAGCATGACTGACATCTAC
Genome GrCh37/hg19
PAM 20bp-NGG - SpCas9
Position chr3:46414382-46414597:+
Version CRISPOR 4.8, 2019-04-12T21:49:22CEST
Results http://crispor.org/crispor.py?batchId=ObBtkMaHaiicT2KjpKmN

#guideId	targetSeq	mitSpecScore	offtargetCount	targetGenomeGeneLocus	Doench '16-Score	Moreno-Mateos-Score	Out-of-Frame-Score	Nomeclature in Study
3forw	CTTTTTATTTATGCACAGGGTGG	58	249	exon:CCR5	51	35	68	(gRNA 1)
173rev	TCAGCCTTTTGCAGTTTATCAGG	72	164	exon:CCR5	35	29	42	
133rev	TAATAATTGATGTCATAGATTGG	69	241	exon:CCR5	40	23	71	(gRNA 2)
61forw	TGACATCAATTATTATACATCGG	64	251	exon:CCR5	56	39	64	(gRNA 3)
167rev	TTTTGCAGTTTATCAGGATGAGG	63	276	exon:CCR5	53	49	31	
67rev	CTTCACATTGATTTTTTGGCAGG	60	292	exon:CCR5	38	0	79	(gRNA 4)
127forw	CCTGCCTCCGCTCTACTCACTGG	58	196	exon:CCR5	47	56	60	
189forw	TCATCCTGATAAACTGCAAAAGG	58	258	exon:CCR5	50	22	44	
111rev	AACACCAGTGAGTAGAGCGGAGG	56	100	exon:CCR5	62	58	62	
66rev	TTCACATTGATTTTTTGGCAGGG	52	410	exon:CCR5	45	22	79	
114rev	ATGAACACCAGTGAGTAGAGCGG	52	210	exon:CCR5	66	49	65	
140forw	TACTCACTGGTGTTCATCTTTGG	51	159	exon:CCR5	30	52	57	
104rev	GTGAGTAGAGCGGAGGCAGGAGG	48	403	exon:CCR5	56	86	67	
160forw	TGGTTTTGTGGGCAACATGCTGG	43	249	exon:CCR5	44	34	33	
107rev	CCAGTGAGTAGAGCGGAGGCAGG	41	210	exon:CCR5	51	40	75	
149forw	GTGTTCATCTTTGGTTTTGTGGG	34	399	exon:CCR5	31	52	51	
148forw	GGTGTTCATCTTTGGTTTTGTGG	33	289	exon:CCR5	40	23	54	
71rev	TTTGCTTCACATTGATTTTTTGG	32	687	exon:CCR5	18	29	73	
100rev	GTAGAGCGGAGGCAGGAGGCGGG	23	791	exon:CCR5	47	66	68	
101rev	AGTAGAGCGGAGGCAGGAGGCGG	20	800	exon:CCR5	48	76	64	

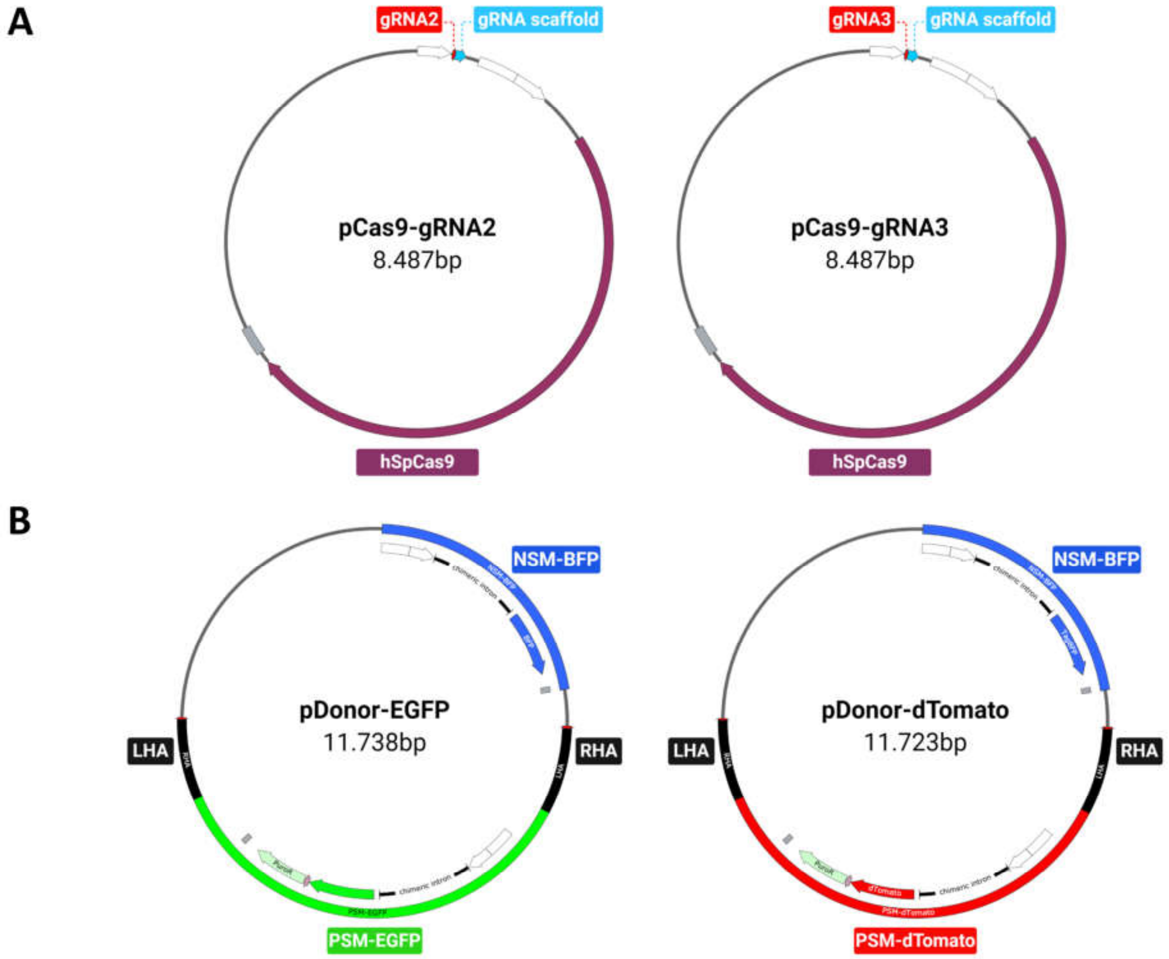

Fig. S1. Schematic diagram of the plasmids used in this study A: For dual targeting, two identical hSpCas9 expressing plasmids were equipped with two different gRNAs (gRNA2 or gRNA3). **B:** The donor plasmids consist of a BFP expression cassette as negative selection module (NSM-BFP) and a positive selection module encoding for EGFP in one donor (PSM-EGFP) and dTomato (PSM-dTomato) in the other, which is flanked by the same sequences homologous for the targeted region (LHA and RHA)

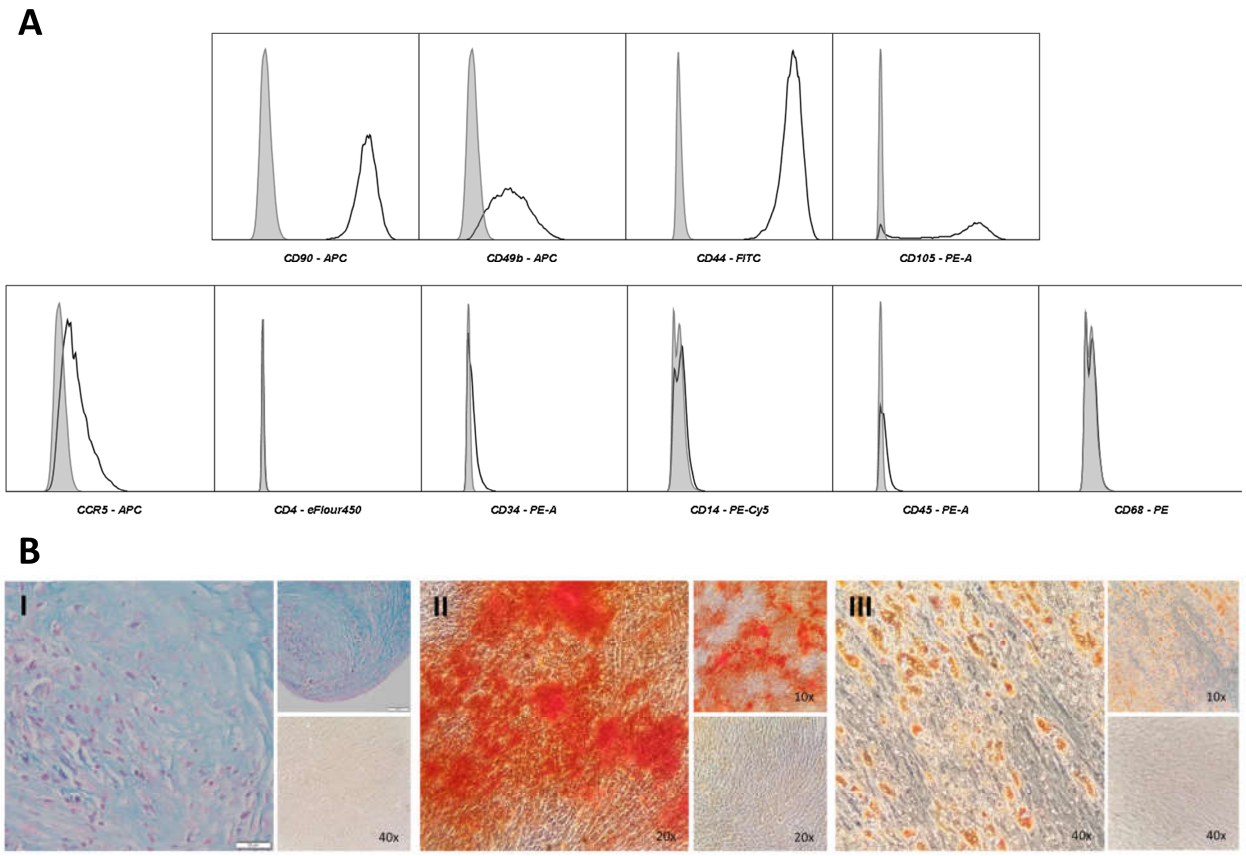

Fig. S2. Characterization of ASC as a multipotent stem cell. A: Immunophenotypic analysis of cell surface profile of freshly isolated ASCs. Cells were stained with fluorescent marker labeled antibodies for CD90, CD49b, CD44, CD105, CCR5, CD4, CD34, CD14, CD45 and CD68. In the histogram the gray filled graph represents the signal of an unstained control, the black line the population is stained with the corresponding antibody. **B:** Multilineage differentiation potential of freshly isolated ASC. Isolated cells were capable of differentiating into (I) chondrogenic (extracellular proteoglycans appear blue in toluidine blue staining), (II) osteogenic (alizarin red staining marks calcium deposits), (III) adipogenic (intracellular lipid vesicles appear red using Oil Red O staining) lineage.

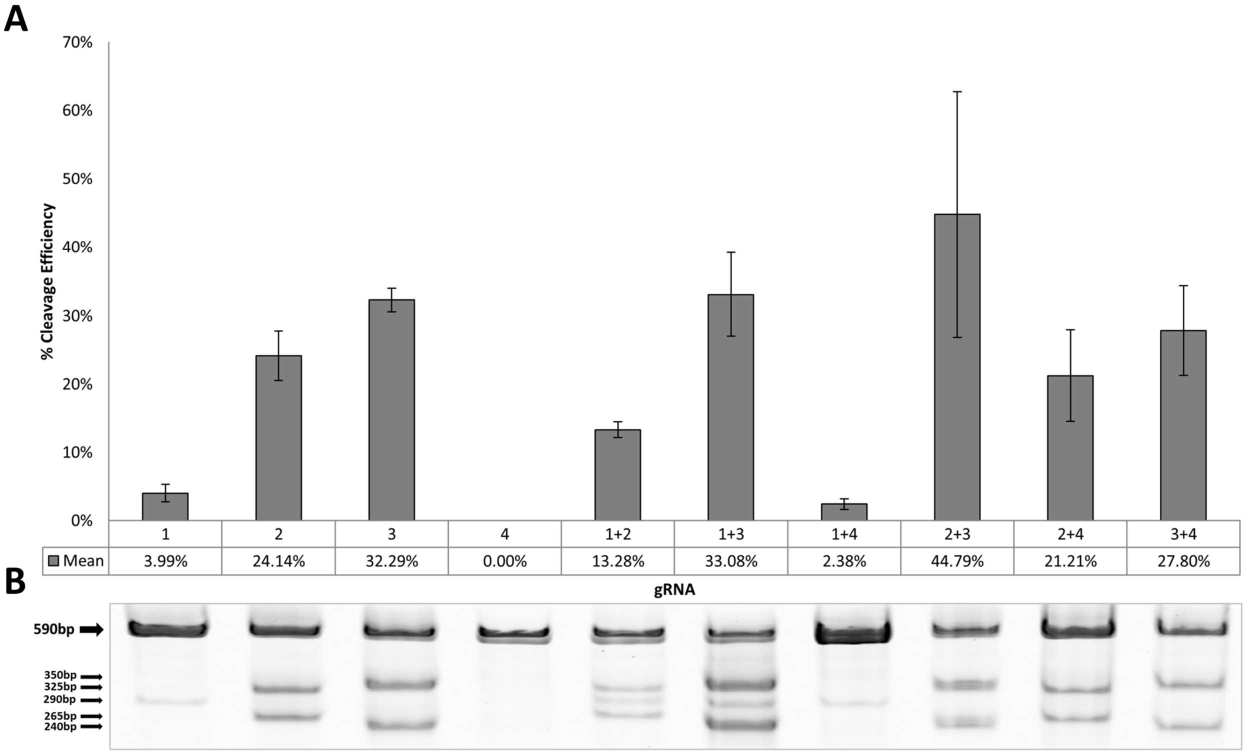

Fig. S3. Identification of the most efficient gRNA and their combinations. A: Four gRNAs (1-4) were cloned into Cas9 expressing Vectors and transfected into HEK 293FT using Lipofection. Additionally dual combinations of the gRNAs were transfected. The targeted site was amplified via PCR and a T7EI assay was performed. Gels were analyzed with ImageJ software and cleavage efficiency was calculated according to band intensity. Transfection and analysis were performed in triplicates. **B:** Representative of one of the triplicate T7EI-Assays visualized on TBE-Gels. Uncut or InDels not detected by the T7 Endonuclease are represented by a 590bp sized band. Bands of lower size represent mutational activity of the different gRNAs (gRNA1 => 290bp; gRNA 2 => 265,325bp; gRNA 3 => 240,350bp; gRNA 4 no bands detectible).

A

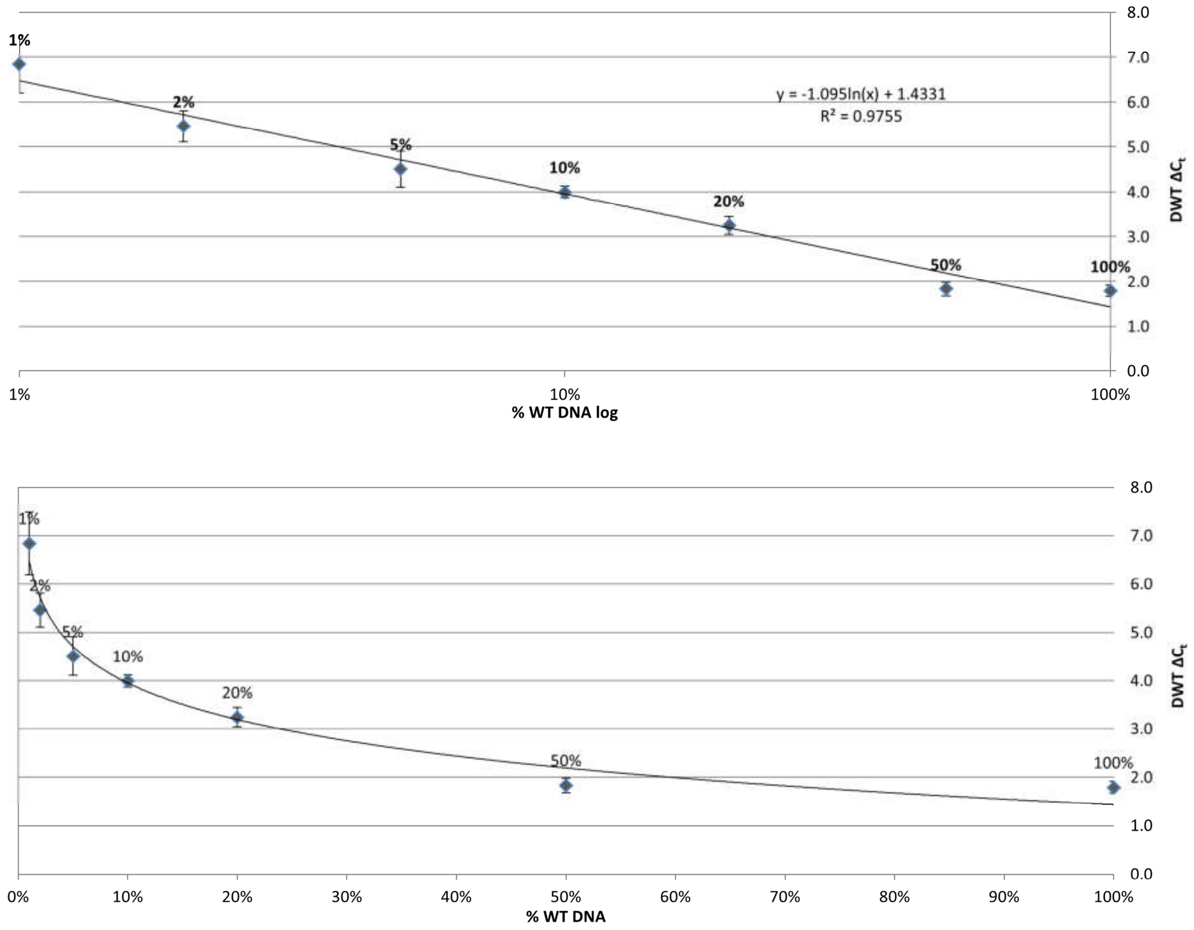

Fig. S4. Standard Curve to determine the fraction of alleles not carrying a Knock-In (DWT) by linear regression from a DWT signal. Genomic DNA extracted from a dually positive clone with integration of the donor on both alleles and WT genomic DNA were mixed at ratios of 1, 2, 5, 10, 20, 50, and 100% WT DNA and subjected to qPCR for the DWT Amplicon. ΔC_t was calculated by normalisation with ß-actin Ct value. The Standard Curve had to be normalized for every cell type analysis individually, by setting the WT DWT ΔC_t to 100% of alleles, not carrying a knock-in. **A:** The % of WT DNA is displayed in a logarithmic manner. **B:** The % of WT DNA is displayed in a linear manner. Due to the logarithmic nature of qPCR, the Ct Value is only sensitive for reflecting the % WT Alleles in the lower ranges. Small changes in the Ct signal reflect massive changes in the calculated % WT DNA in populations with an high fraction of WT alleles.

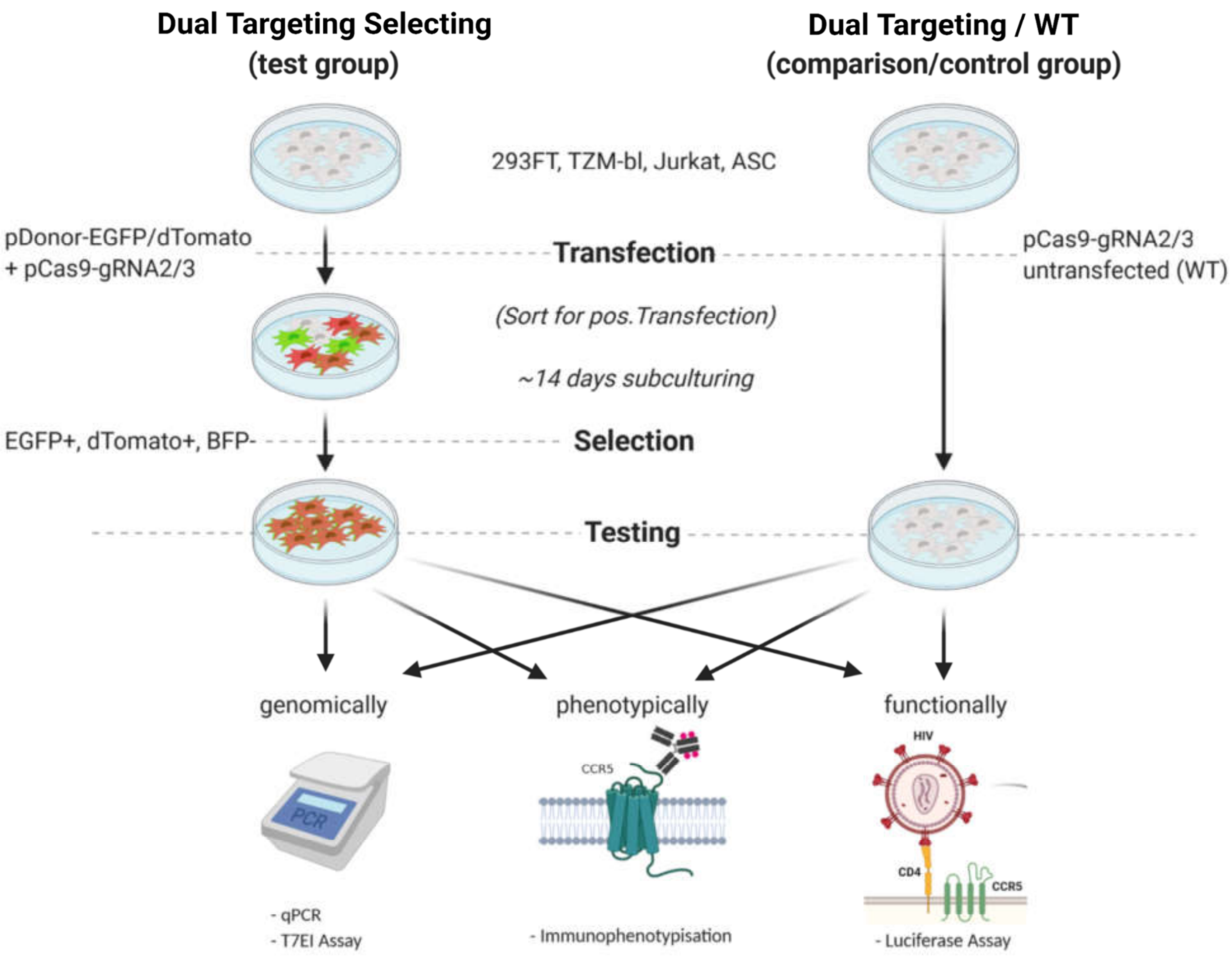

Fig. S5. Experimental Outline / Graphical Abstract
